# Advantages of transformer and its application for medical image segmentation: a survey

**DOI:** 10.1186/s12938-024-01212-4

**Published:** 2024-02-03

**Authors:** Qiumei Pu, Zuoxin Xi, Shuai Yin, Zhe Zhao, Lina Zhao

**Affiliations:** 1 School of Information Engineering, Minzu University of China, Beijing, 100081 China; 2 CAS Key Laboratory for Biomedical Effects of Nanomaterials and Nanosafety Institute of High Energy Physics, Chinese Academy of Sciences, Beijing, 100049 China; 3 The Fourth Medical Center of PLA General Hospital, Beijing, 100039 China

**Keywords:** Deep learning, Transformer, Medical image, Segmentation, Codec

## Abstract

**Purpose:**

Convolution operator-based neural networks have shown great success in medical image segmentation over the past decade. The U-shaped network with a codec structure is one of the most widely used models. Transformer, a technology used in natural language processing, can capture long-distance dependencies and has been applied in Vision Transformer to achieve state-of-the-art performance on image classification tasks. Recently, researchers have extended transformer to medical image segmentation tasks, resulting in good models.

**Methods:**

This review comprises publications selected through a Web of Science search. We focused on papers published since 2018 that applied the transformer architecture to medical image segmentation. We conducted a systematic analysis of these studies and summarized the results.

**Results:**

To better comprehend the benefits of convolutional neural networks and transformers, the construction of the codec and transformer modules is first explained. Second, the medical image segmentation model based on transformer is summarized. The typically used assessment markers for medical image segmentation tasks are then listed. Finally, a large number of medical segmentation datasets are described.

**Conclusion:**

Even if there is a pure transformer model without any convolution operator, the sample size of medical picture segmentation still restricts the growth of the transformer, even though it can be relieved by a pretraining model. More often than not, researchers are still designing models using transformer and convolution operators.

## Introduction

Medical image segmentation is a significant study area in computer vision, to classify medical pictures at the pixel level and then precisely segment the target item. Segmentation datasets are created from unimodal or multimodal pictures obtained by professional medical equipment such as magnetic resonance imaging (MRI), computed tomography (CT), and ultrasonography (US). Traditional nondeep learning medical picture segmentation approaches depend mostly on thresholding [1], region growth [2], border detection [3], and other techniques. To produce superior segmentation results, picture features must be manually extracted before segmentation. The feature extraction methods for various datasets are frequently diverse, and some professional experience is necessary [4–6]. The deep learning-based segmentation approach can automatically learn the feature that represents the picture, but it requires a high-performance computer and takes a long time to train the network.

With the continual advancement of computer equipment such as Graphic Processing Units (GPU) in recent years, training most deep learning models is no longer constrained. At present, the segmentation model-based convolutional neural network (CNN) is extensively employed in a variety of medical picture segmentation applications [7, 8], including tumor segmentation [9], skin lesion region segmentation [10], left and right ventricular segmentation [11], and fundus blood vessel segmentation [12]. U-Net [13] is one of the most extensively utilized models. Through skip connections, U-Net integrates the multiscale detail information in the picture downsampling process with the global properties of low-resolution images. This encoder–decoder design, which combines information at multiple scales, considerably enhances segmentation model performance and is frequently utilized in the field of medical picture segmentation. However, CNN can only employ very tiny convolution kernels to balance model accuracy and computational complexity, limiting it to a relatively restricted perceptual domain. It excels at obtaining local characteristics but falls short of capturing long-distance dependencies. Similar to domains such as autonomous driving, satellite image analysis, and pedestrian recognition, medical image analysis also encounter challenges like unclear boundaries [14], low contrast, varying object sizes, and complex patterns. Addressing these challenges often hinges on incorporating a broader contextual perspective, encompassing global background information.

**Fig. 1:**
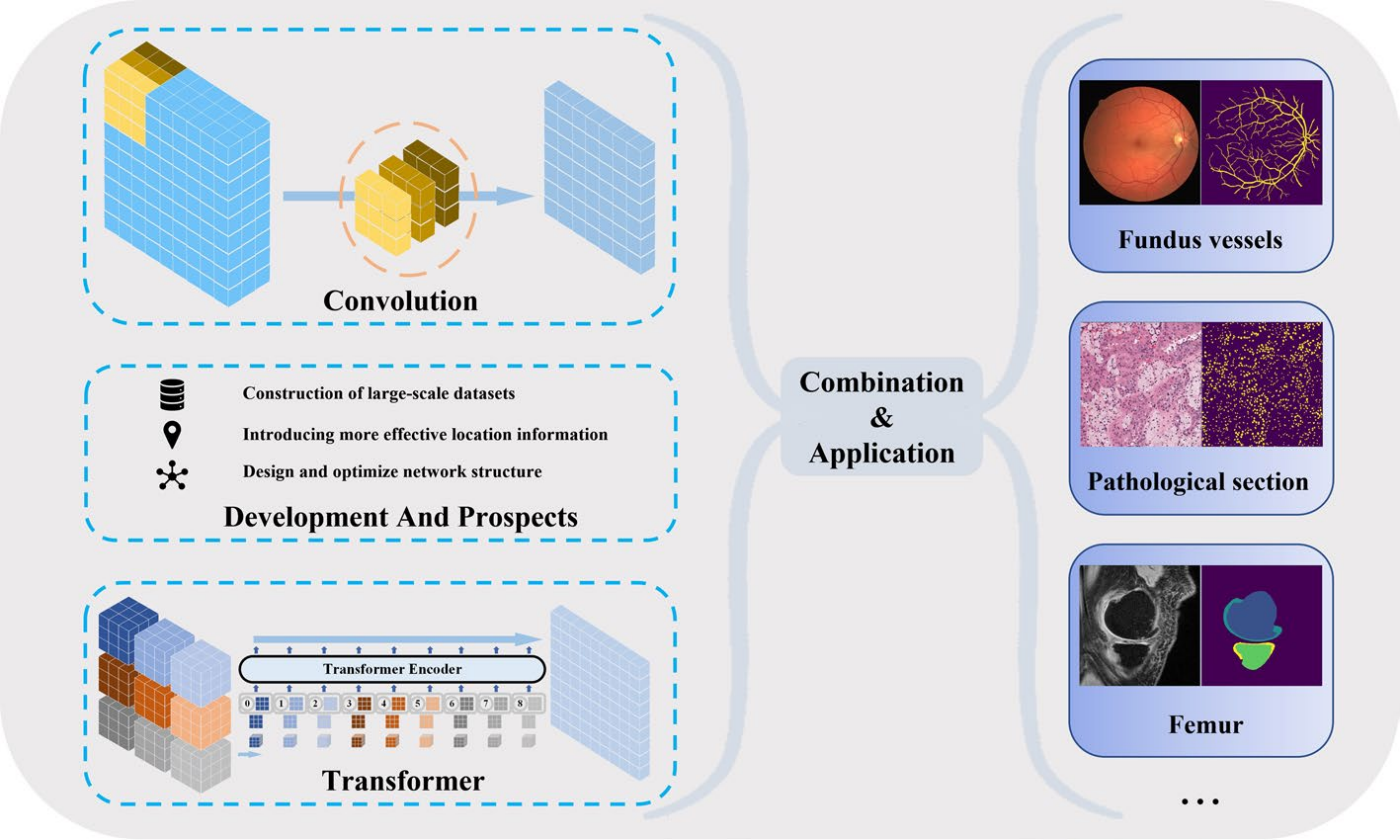
Combining CNN with Transformer improves various medical image segmentation tasks

**Fig. 2:**
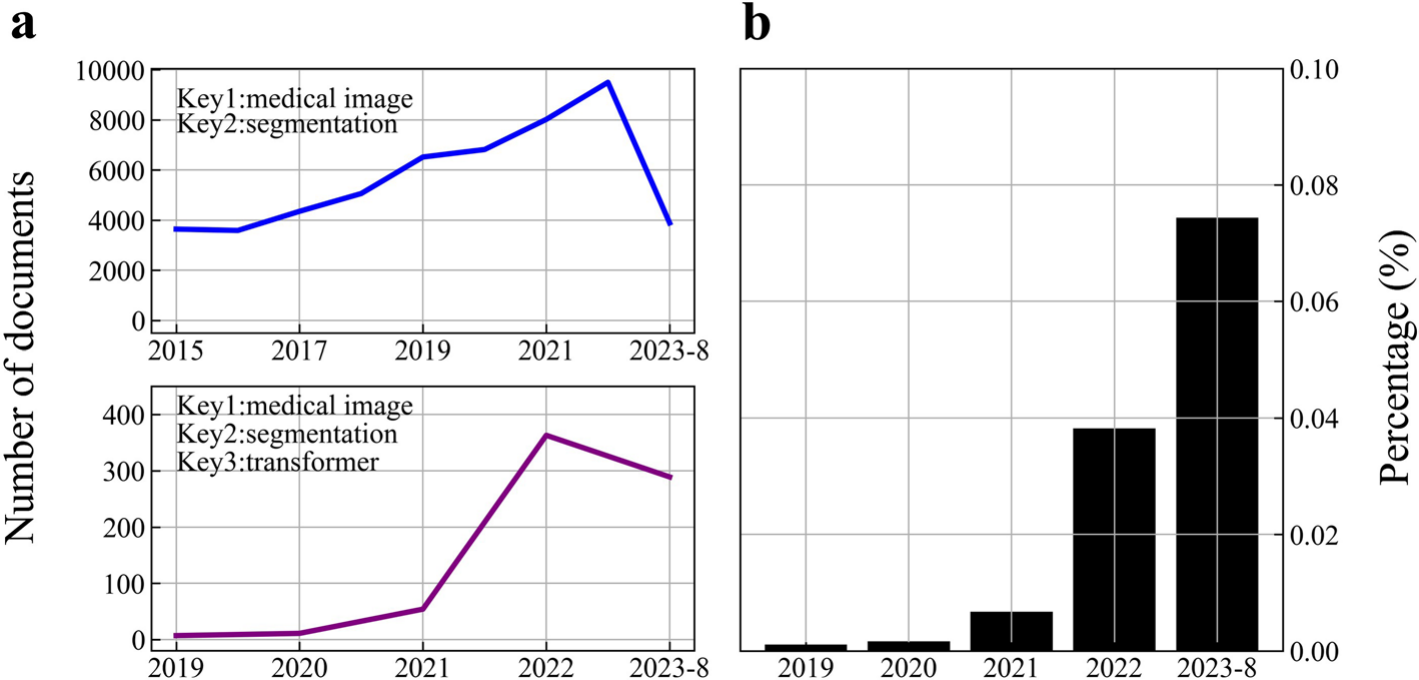
Using web of science to retrieve and statistically analyze literature. **a** Statistics of literature quantity for two sets of keywords. **b** The proportion of literature related to transformers in medical image segmentation literature

Through the self-attention process, the popular transformer [15] in machine translation and sentiment analysis may gather global context information. Following the successful application of pure transformer architecture to the field of computer vision by ViT [16], an increasing number of transformer-based models have been developed to optimize medical picture segmentation approaches (Fig. 1). We analyzed articles published in the last 5 years on web of science using two sets of keywords, as shown in Fig. 2. The first set of keywords included ’medical image’ and ’segmentation,’ while the second set consisted of ’medical image,’ ’segmentation,’ and ’transformer.’ As depicted in Fig. 2a, medical image segmentation has consistently remained a prominent research area, with nearly 5000 publications each year. The introduction of Vision Transformer (ViT) in 2020 marked the beginning of increased interest in using transformers for medical image segmentation, leading to rapid growth. The number of articles surged by more than 400%, particularly in 2021 and 2022. The finding from Fig. 2b also demonstrates the growing proportion of the second group, which is a subset of the first group of literature. These statistical findings underscore the significant potential of transformers in the field of medical image segmentation.

Currently, several review articles have summarized literature related to Transformers in the field of medical image segmentation. However, these reviews are often context-specific, focusing on different medical applications, such as categorization based on disease types [17], task-oriented summaries [18, 19], or aggregations based on specific medical images or diseases [20–22]. The synthesis and categorization based on network structures are crucial for optimizing deep learning models for diverse tasks, yet research in this domain is currently limited. This paper explores recent advancements in research on medical image segmentation tasks using transformer and encoder–decoder structural models. It provides a comprehensive study and analysis of relevant deep learning network structures, aiming to further uncover the potential of transformer and encoder–decoder structural models in medical image segmentation tasks. The objective is to guide researchers in designing and optimizing network structures for practical applications.

In the "Basic model structure" section, we will delve into the pertinent information regarding the encoder–decoder structure and transformer. "Medical Image Segmentation Method Based on Transformer" section will present a comprehensive summary of transformer segmentation methods, considering four perspectives: Transformer in the encoder, Transformer in the codec, Transformer in the skip connections, and the application of the pure Transformer structure. Each subsection within "Medical Image Segmentation Method Based on Transformer" section sequentially elaborates on the optimization and enhancement details of various models. Detailed evaluation metrics for medical image segmentation are outlined in "Evaluation Indicators" section. "Dataset" section systematically organizes the medical image segmentation datasets suitable for reproducing model results. Finally, "Summary and Outlook" will encapsulate the conclusion and provide insights for future developments.

## Basic model structure

### Codec structure in medical image segmentation

**Fig. 3:**
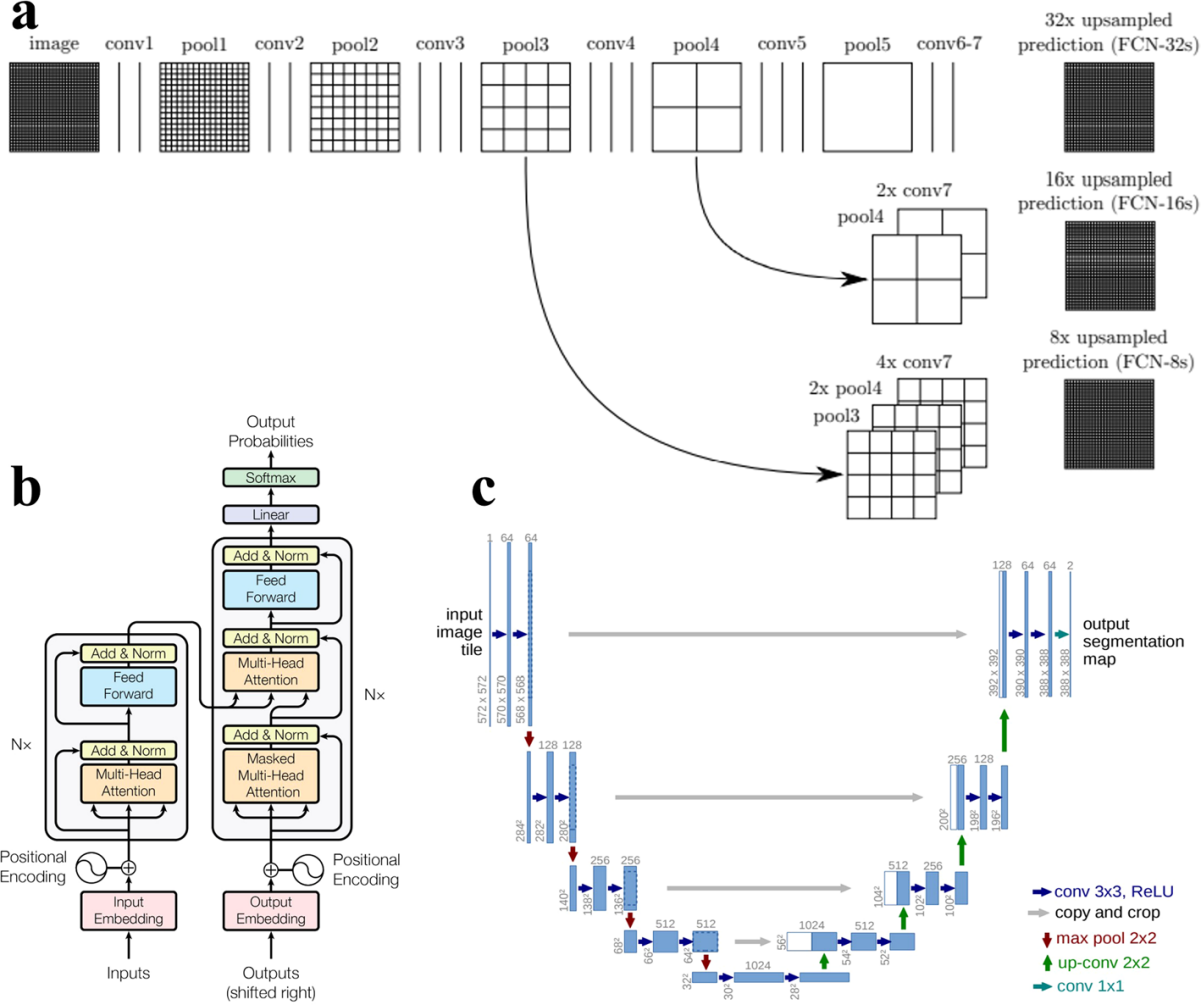
Codecs and transformer architectures. **a** FCN network structure [24]. **b** A transformer block [15].** c** Classical U-Net architecture [13]

Because of the codec structure, the entire network is made up of an encoder module and a decoder module. The encoder is primarily responsible for extracting features from the input, while the decoder is responsible for additional feature optimization and job processing on the encoder’s output. Hinton [23] initially presented this architecture in Science in 2006, with the primary goal of compressing and denoising rather than segmentation. The input is an image, which is downsampled and encoded to generate features that are smaller than the original picture, a process known as compression, and then sent through a decoder, which should restore the original image. For each image, we need to save only one feature and one decoder. Similarly, this concept may be applied to picture denoising, which involves adding fake noise to the original image during the training stage and then inserting it into the codec to restore the original image. This concept was then used for the picture segmentation problem. Encoders in medical picture segmentation tasks are often based on existing backbone networks such as VGG and ResNet. The decoder is often constructed to meet the job requirements, labeling each pixel progressively by upsampling. In 2015, Long introduced a groundbreaking approach called the Fully Convolutional Neural Network (FCN) [24] for semantic segmentation, as illustrated in Fig. 3a. The FCN converts the CNN’s final fully connected layer to a convolutional layer and merges features from multiple layers using simple skip connections. Finally, deconvolution restoration is used to achieve end-to-end picture segmentation. The FCN segmentation results are far from comparable to the manual segmentation results because of upsampling and fusing features of various depths. There are still many locations with segmentation faults, particularly around the edges. At the same time, the architecture of the FCN’s single-path topology makes it impossible to preserve meaningful spatial information in upsampled feature maps and lacks network space consistency.

One of the most often used models in medical picture segmentation tasks is the U-Net model, which is built on the principle of FCN to extract multiscale features. As shown in Fig. 3c, the U-Net network initially executes four downsampling operations on the input picture to extract image feature information, followed by four sets of upsampling. To assist the decoder in repairing the target features, a skip connection with a symmetric structure is inserted between the downsampling and upsampling procedures. On the right, the output of the downsampled convolutional block is concatenated with the input of the deconvolutional block with the same depth. The initial difference between U-Net and FCN is that U-Net is extremely symmetric, and the decoder is very similar to the encoder, but FCN’s decoder is quite simple, simply utilizing a deconvolution operation and no convolutional structure thereafter. The skip connection is the second distinction. FCN uses summation, whereas U-Net employs concatenation. In MICCAI 2016, Cicek et al. expanded 2D U-Net to 3D U-Net and utilized 3D U-Net [25] to segment dense collective pictures from sparse annotations. nnU-Net [26] is an adaptive framework for any dataset based on U-Net, 3D U-Net, and U-Net Cascade. It can automatically adjust all hyperparameters according to the properties of a given dataset without human intervention throughout the process, achieving advanced performance in six well-recognized segmentation challenges. U-Net has quickly become an essential network model in medical picture segmentation due to its great performance and unique topology.

### Transformer

Benjio’s team proposed the attention mechanism in 2014, and it has since been widely used in various fields of deep learning, such as computer vision to capture the receptive field on an image, or NLP to locate key tokens or features. The multihead attention mechanism, position encoding, layer regularization [27], feedforward neural network, and skip connection are the main components of the encoder. The decoder differs from the encoder in that it includes an additional masked multihead attention module in the input layer, but the rest of the components are the same. The self-attention mechanism is an important part of the transformer, and its unique design allows it to handle variable-length inputs, capture long-distance dependencies, and seq2seq.

1 $$Attention\left( {q,k,v} \right) = softmax\,\left( {\frac{{qk^{T} }}{{\sqrt {d_{k} } }}} \right)v,{\text{ }}$$

where *q*, *k,* and *v* are vectors of input *X* after linear mapping, and $d_k$ is the dimension of the vector. After parallel computing, the multihead attention mechanism extracts features from multiple self-attention mechanism modules and concatenates them in the channel dimension. Various groups of self-attention mechanisms can learn various types of feature representations from subspaces at various locations.

2 $$MultiHead\,\left( {Q,K,V} \right) = Concat\,\left( {Attention\,\left( {QW_{i}^{Q} ,KW_{i}^{K} ,VW_{i}^{V} } \right), \cdots ,Attention\,\left( {QW_{H}^{Q} ,KW_{H}^{K} ,VW_{H}^{V} } \right)} \right)W^{o} ,$$

where *Q*, *K*, and *V* are matrices made up of multiple *q*, *k*, and *v* vectors. $i = 1,2,\dots ,H; d_k =d_v = d_{model}/H$; $W_i^Q$ and $W_i^K$ are matrices in the form of ($d_{model}$, $d_k$), $W_i^V$ is matrices in the form of ($d_{model}$, $d_v$), and the three matrices are parameter matrices used to map input.

The decoder’s masked multihead attention mechanism takes into account the fact that during the testing and verification phases, the model can only obtain information before the current position. To avoid the model’s reliance on information after the current position in the testing phase, the information after the current position is masked in the training phase, ensuring that only information before the position is used to infer the current result. Because of the unique design of self-attention, it is insensitive to sequence position information, which is important in both natural language processing and computer vision tasks, so position information must still be incorporated into transformers. Transformers frequently use sine and cosine functions to learn position information.

Layer regularization overcomes batch regularization’s shortcoming of making it difficult to handle tasks with variable input sequences. It shifts the scope of regularization from across samples to within the same sample’s hidden layer, so that regularization is independent of input size. Skip connection is a widely used technique for improving the performance and convergence of deep neural networks, as it alleviates the convergence of nonlinear changes via the linear components propagated through the neural network layers. If the patch is too small in the transformer, there will be a false-gradient explosion or disappearance.

### Vision transformer

**Fig. 4:**
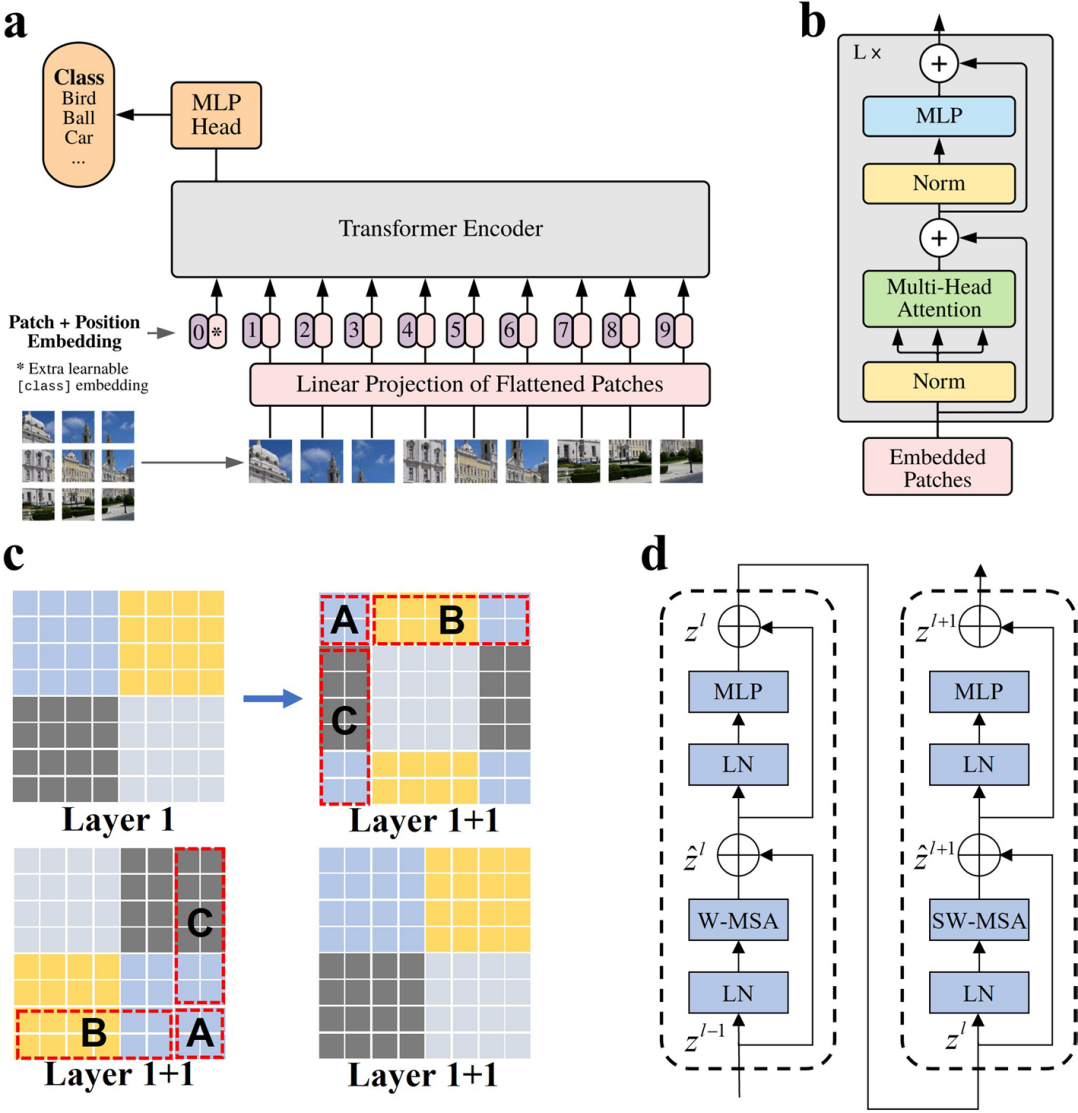
Key components of the ViT and Swin Transformer. **a** The ViT architecture, showcases the transformation of input feature maps into patches, followed by linear mapping and processing through the Transformer. The result undergoes classification via an MLP. **b** The details of the ViT encoder, emphasizing the integration of multihead attention modules. **c** The feature map evolution in Swin Transformer during W-MSA and SW-MSA computation, highlighting the cyclic shift operation for integrating shifted window feature maps. **d** Swin Transformer Block, outlining its computational process

In 2020, Google introduced the ViT [16], a model that leverages the transformer architecture for image classification. ViT innovatively partitions input images into multiple patches, each measuring 16x16 pixels. These patches are then individually transformed into fixed-length vectors and integrated into the Transformer framework, as illustrated in Fig. 4a. Subsequent encoder operations closely mirror the original Transformer architecture, as depicted in Fig. 4b. While not the pioneer in exploring transformers for computer vision, ViT stands out as a seminal contribution due to its “simple” yet effective model, robust scalability (larger models demonstrating superior performance), and its groundbreaking influence on subsequent research in the field. With sufficiently large pretraining datasets, ViT surpasses CNN, overcoming the limitation of transformers lacking inductive bias and showcasing enhanced transfer learning capabilities in downstream tasks.

In March 2021, Microsoft Research Asia proposed a universal backbone network named Swin Transformer [28]. The Swin Transform Block is constructed differently from ViT, employing Window Multihead Self-Attention (W-MSA) and Shifted Window Multi-head Self-Attention (SW-MSA). When computing W-MSA, an 8x8 feature map is divided into 2x2 patches, each with a size of 4x4. For SW-MSA, the entire set of patches is shifted by half the patch size, creating a new window with non-overlapping patches. This approach introduces connections between adjacent non-overlapping windows, significantly increasing the receptive field. However, it also raises the issue of increasing the number of patches within the window from 4 to 9. To maintain the original patch count, the authors employ a cyclic shift operation, as illustrated in Fig. 4c. W-MSA calculates attention within each window, while SW-MSA utilizes global modeling, akin to ViT, to establish long-distance dependencies. As depicted in Fig. 4d, Swin Transformer’s unique design not only introduces local feature extraction capabilities similar to convolution but also substantially reduces computation. Swin Transformer achieves state-of-the-art performance in machine vision tasks such as image classification, object detection, and semantic segmentation.

## Medical image segmentation method based on transformer

Prior to the application of transformer to the field of medical image segmentation, segmentation models such as FCN and U-Net performed well in various downstream image segmentation tasks. Researchers have used various methods to improve the U-Net model to meet the needs of different tasks and data, and a series of variant models based on the U-Net model have appeared; for example, 3D U-Net [25], ResUNet [29], U-Net++ [30], and so on. However, since the introduction of ViT, an increasing number of researchers have focused on the attention mechanism, attempting to apply it locally or globally in complex network structures to achieve better results. By incorporating a transformer module during encoder downsampling, TransUNet [31] outperforms models such as V-Net [32], DARR [33], U-Net [13], AttnUNet [34], and ViT [16] in a variety of medical applications, including multiorgan segmentation and heart segmentation. TransUNet, like U-Net, has become a popular network for medical image segmentation. Because of the complexities of medical image segmentation tasks, high-quality manually labeled datasets can only be produced on a small scale. To achieve better performance on medical image datasets, it is necessary to continuously optimize the application of transformer in the encoder/decoder network. Following that, this paper will discuss transformer-based medical image segmentation methods based on model optimization position.

### Transformer encoder structure

**Fig. 5:**
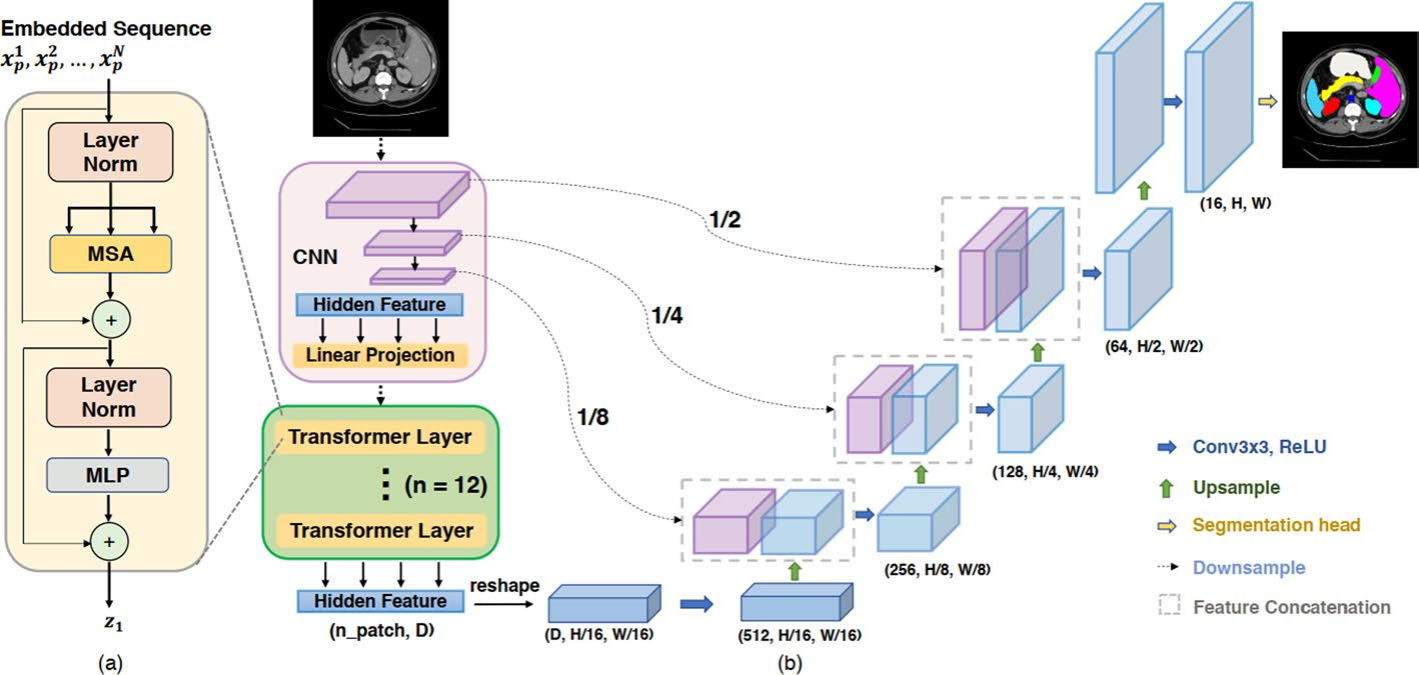
TransUnet applied transformer structure to medical image segmentation firstly [31]. **a** schematic of the Transformer layer; **b** architecture of the proposed TransUNet

TransUNet, depicted in Fig. 5, stands as the pioneering application of the transformer model in the realm of image segmentation. The authors serialize the feature map obtained through U-Net downsampling and then process the serialized features with a block made up of 12 original transformer layers. The benefits of long-distance dependencies can be obtained using transformers to capture global key features. The experimental results show that TransUNet outperforms the previous best model, AttnUNet, on the Synapse dataset. TransBTS [35] replaces 2D CNNS with 3D CNNS and uses a structural design similar to TransUNet to achieve 3D multimodal brain tumor segmentation in MRI imaging. Similar to TransBTS, the UNETR [36] employs the same 12 transformer blocks in its encoder. However, UNETR differs in that it utilizes the outputs of the 3rd, 6th, 9th, and 12th transformer blocks as inputs for four downsampling convolutional neural network modules in the encoder. UNETR demonstrates excellent performance in both BTCV [37] and MSD [38], two 3D image segmentation tasks. Furthermore, Swin UNETR [39] goes a step further by replacing the Transformer blocks in UNETR with Swin Transformer blocks, achieving superior results on the BraTS2021 dataset compared to nnU-Net, SegResNet, and TransBTS. AFTer-UNet [40] employs an axial fusion transformer encoder between CNN encoder and CNN decoder to integrate contextual information across adjacent slices. The axial Fusion transformer encoder calculates attention along the axial direction and within individual slices, reducing computational complexity. This approach significantly outperforms models like CoTr and SwinUnet on multiorgan segmentation datasets, including BCV [41], Thorax-85 [42], and SegTHOR [43].

In general, most methods for dealing with 2D image segmentation can also be used to deal with continuous video data, as long as the video data are input as a 2D image frame by frame. The cost of this is that we cannot fully exploit the time continuity of the video data. Zhang et al. [44] created an additional convolution branch based on TransUNet to extract the features of the previous frame data, and then combined the results of the downsampling of the two parts with the results of the upsampling via the skip connection to achieve a better video data segmentation effect. X-Net [45] extends U-Net by introducing an additional Transformer-based encoder–decoder branch, facilitating information fusion across branches through skip connections. Zhang et al. proposed a new architecture called TransFuse, which can run convolution-based and pure transformer-based encoders in parallel and then fuse the features from the two branches together to jointly predict segmentation results via the BiFusion module, greatly improving the model’s inference speed [46]. This work adds a new perspective to the use of transformer-based models by investigating whether a network using only transformers and no convolution can perform better segmentation tasks.

The primary goal of the self-attention mechanism is to model the long-distance dependence between pixels to obtain global context information. On the other hand, convolution produces feature maps at various scales that frequently contain complex information. Before the appearance of ViT, researchers discovered numerous effective methods for expanding the convolution receptive field using convolution. Dilated convolutions are the most well-known of these, and DeepLabV3 [47] uses dilated spatial pyramid pooling to great effect, while CE-Net [48] captures multiscale information using dense dilated convolutions and residual multikernel pooling. As a result, taking into account global context information and multiscale information is a very effective method. Yuanfeng Ji [49] et al. proposed MCTrans, a self-attention transformer module and a cross-attention transformer module. The self-attention transformer module performs pixel-level context modeling at multiple scales. To ensure intraclass consistency and interclass discrimination, the cross-attention transformer module is used to learn the corresponding semantic relationship of different categories, that is, the difference between feature expressions of different classes and the connection between feature expressions of different classes. DC-Net [50] also reflects the emphasis on multiscale features in this model. The authors create a Global Context Transformer Encoder (GCTE) and a module for Adaptive Context Fusion (ACFM). GCTE connects the transformer encoder to the back of CNN down-sampling, serializes the multiscale features obtained by CNN and the input image, and then obtains a better feature representation via the transformer encoder. The ACFM is made up of four cascaded feature decoding blocks, each with two 1$\times$1 convolutions and a 3$\times$3 deconvolution. The adaptive weight $\omega _i$ is converted by the authors into adaptive spatial weight (APW) and adaptive channel weight (ACW). The ACFM can better fuse context information and improve decoder performance using the two weight parts of the APW and ACW.

Although transformers have achieved outstanding results in a variety of downstream medical image tasks, it is undeniable that they have more parameters to train than convolutional models. As a result, how to optimize the model using global context information obtained by the transformer to meet the requirements of lightweight tasks for model size and inference speed has become a hot topic in research. SA-Net [51] was proposed in early transformer-related research to reduce the number of parameters in CNN and transformer using a random ranking algorithm. The sandwich parameter-shared encoder structure [52] was investigated by Reid M et al. In the field of medical image segmentation, the CoTr model [53] was proposed by Xie Y et al. The encoder structure was created by combining the bridge structure DeTrans, which was made up of the MS-DMSA layer and only focused on a small set of key sampling locations around the reference location, with CNN, which greatly reduced the time and space complexity. TransBridge [54] employs a bridge structure similar to CoTr, but adds a shuffle layer and group convolution to the transformer’s embedding part to reduce the number of parameters and the length of the embedding sequence. The experimental results show that after 78.7% parameter reduction, on the EchoNet-Dynamic dataset, TransBridge outperforms CoTr, ResUNet [29], DeepLabV3 [55], and other models.

### Transformer codec structure

TransUNet demonstrated the importance of transformers in encoders, and the symmetries of encoder–decoder architectures make it simple to extend transformers to decoder architectures. U-Transformer [56] uses the Multihead Cross-attention Module (MHCA) to combine the high-level feature maps with complex abstract information and the high-resolution feature maps obtained through the skip connection in each splicing process of upsampling and skip connection, which is used to suppress the irrelevant regions and noise regions of the high-resolution feature maps. The feature map obtained by convolution is expanded pixel by pixel as a transformer patch in the encoder section, and then a single transformer layer is used to extract global context information. Luo C et al. [57] improved the use of transformer in encoders based on the TransUNet and U-Transformer. To build the UCATR model, a block of 12 transformer layers is used to replace the single MultiHead self-attention in the U-Transformer. The experimental results show that the UCATR model can recover more refined spatial information than the original TransUNet and U-Transformer. SWTRU [58] proposes a novel Star-shaped Window self-attention mechanism to be applied in the decoder structure and introduces the Filtering Feature Integration Mechanism (FFIM) to integrate and reduce the dimensionality of the fused multilayer features. These improvements result in a better segmentation effect in CHLISC [59, 60], LGG [61, 62], and ISIC2018 [63]. Since in most vision tasks the visual dependencies between regions nearby are usually stronger than those far away, MT-UNet [64] performs local self-attention on fine-grained local context and global self-attention only on coarse-grained global context. When calculating global attention maps, axial attention [65] is used to reduce the amount of calculation, and further introduce a learnable Gaussian matrix [66] to enhance the weight of nearby tokens. MT-UNet performs better than models such as ViT and TransUNet on the Synapse and ACDC datasets.

Although transformers have done much useful work in medical image segmentation tasks, training and deploying transformer-based models remains difficult due to a large amount of training time and memory space overhead. To reduce the impact of the sequence length overhead, one common method is to use the feature maps obtained by downsampling as the input sequence rather than the entire input image. High-resolution images, on the other hand, are critical for location-sensitive tasks such as medical image segmentation, because the majority of false segmentations occur within the region of interest’s boundary range. Second, in medical image data with small data volumes, transformers have no inductive bias and can be infinitely enlarged.

Gao Y et al. [67] combined the benefits of convolution and the attention mechanism for medical image segmentation, replacing the last layer of convolution with a transformer module in each downsampling block, avoiding large-scale transformer pretraining while capturing long-distance correlation information. At the same time, to extract the detailed long-distance information on the high-resolution feature map, two projections are used to project the K and V (*K* and $V \in R_{n \times d}$) into low-dimensional embedding (*K* and $V \in R_{k \times d}$, $k = hw \ll n$), where *h* and *w* are the reduced sizes of the feature map after subsampling, which reduces the overall complexity from $O(n^2)$ to *O*(*n*). In addition, the authors also learn the content–location relationship in medical images using relative position encoding in the self-attention module. Valanarasu J et al. [68] proposed an MedT model based on a gated location-sensitive attention mechanism, which allowed the model to perform well on smaller datasets during training. Feiniu Yuan et al. [69] introduced CTC-Net, a synergistic network that combines both CNN and transformer for medical image segmentation. This approach involves feature extraction through both a CNN encoder and a Swin Transformer encoder, followed by feature fusion facilitated by an Feature Complementary Module (FCM) incorporating channel attention and spatial attention mechanisms.

### Transformer in skip connections

The mechanism of skip connections was initially introduced in U-Net, aiming to bridge the semantic gap between the encoder and decoder, and has proven to be effective in recovering fine-grained details of the target objects. Subsequently, UNet++ [30], AttnUnet [34], and MultiResUNet [70] further reinforced this mechanism. However, in UCTransUnet [71], the authors pointed out that skip connections in U-Net are not always effective in various medical image segmentation tasks. For instance, in the GlaS [72] dataset, a U-Net model without skip connections outperforms the one with skip connections, and using different numbers of skip connections also yields different results. Therefore, the authors considered adopting a more suitable approach for feature fusion at different depths. They replaced the simple skip connections in U-Net with the CTrans module, consisting of multiscale Channel Cross fusion with Transformer (CCT) and Channel-wise Cross-Attention (CCA). This modification demonstrated competitive results on the GlaS and MoNuSeg [73] datasets.

### Pure transformer structure

Researchers have attempted to use transformer as a complete replacement for convolution operators in codec structures due to its significant advantage in global context feature extraction. Karimi D et al. [74] pioneered the nonconvolutional deep neural network for 3D medical image segmentation, demonstrating through experiments that a neural network fully composed of transformer modules can achieve segmentation accuracy superior to or comparable to the most advanced CNN model 3D UNet++ [30].

**Fig. 6:**
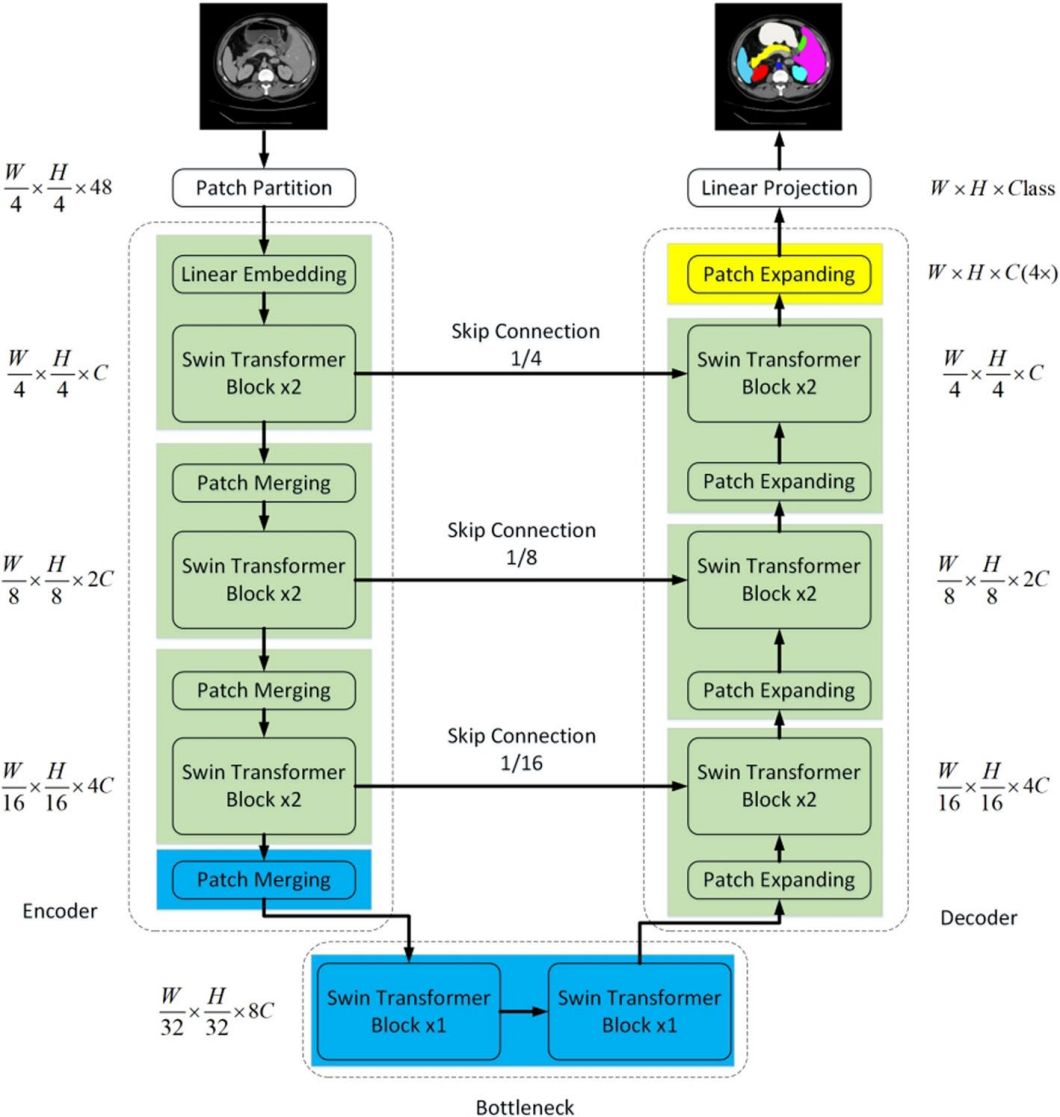
The Swin-Unet structure [75]

Based on the Swin Transformer, Cao H et al. [75]created Swin-Unet, a pure transformer model similar to U-Net. The model employs two consecutive Swin Transformer blocks as a bottleneck, which are then assembled in a U-Net-like configuration. The structure of Swin-Unet is shown in Fig. 6. By comparing Swin-Unet with V-Net [32], DARR [33], ResUnet [29], AttnUnet [34] and TransUnet [31] on two datasets of Synapse and ACDC, the authors obtained significantly better performance than other models. Swin-PANet [76] is a dual supervision network structure proposed by Zhihao Liao et al. Swin-PANet is made up of two networks: a prior attention network and a hybrid transformer network. The prior attention network applies the sliding window-based subattention mechanism to the intermediate supervision network, whereas the hybrid transformer network aggregates the features of the jump connection and the prior attention network and refines the boundary details. GlaS [72] and MoNuSeg [73] yield better results. DS-TransUNet [77] is constructed upon the SwinTransformer framework and enhances feature representation with a dual-scale encoder. More precisely, the approach employs medical images segmented at both large and small scales as inputs to the encoder. This allows the model to effectively capture coarse-grained and fine-grained feature representations.

These models demonstrate the Swin Transformer’s utility for medical image datasets. Because the Swin Transformer is more lightweight and suitable for medical image segmentation tasks than transformers that require large amounts of data pretraining in NLP, further investigating its application can help overcome the challenge of limiting model progress in medical image datasets.

## Evaluation indicators

The objective evaluation of the performance of medical image segmentation algorithms is essential for their practical application in diagnosis. The segmentation results must be assessed both qualitatively and quantitatively. For segmentation tasks with multiple categories, let *k* be the number of classes in the segmentation result, $p_{ij}$ be the total number of pixels whose class *i* is predicted to be the total number of class *j*, and $p_{ii}$ be the total number of pixels whose class *i* is predicted to be the total number of class *i*. When $k=2$, we can divide the results of a segmentation task with only two classes into four categories: True positive (TP) indicates that both the observed and predicted data classes are correct. True negative (TN) indicates that both the actual and predicted data classes are incorrect. The term false positive (FP) refers to when the actual data class is false while the predicted data class is true. The term false negative (FN) denotes that the actual data class is true while the predicted data class is false. The following are examples of commonly used evaluation metrics.

The F1 score, or F-measure, is a metric used in binary classification analysis, representing the harmonic mean of precision and recall. Precision is the ratio of true positive results to all identified positive results, while recall is the ratio of true positive results to all actual positive instances. By combining precision and recall in a single metric, the F1 score provides a balanced measure of a test’s accuracy. It ranges from 0 to 1, with 1 indicating perfect precision and recall, and 0 if either precision or recall is zero.

3 $$\begin{aligned}{} & {} {\text {Precision}}=\frac{TP}{TP+FP} \end{aligned}$$

4 $$\begin{aligned}{} & {} {\text {Recall}}=\frac{TP}{TP+FN} \end{aligned}$$

5 $$\begin{aligned}{} & {} {{\text {F}}_{1}}=2\times \frac{Precision\times Recall}{Precision+Recall} \end{aligned}$$

The prediction results are evaluated using pixel accuracy (PA), which stands for the proportion of total pixels classified correctly over the total number of pixels of original samples. The PA value is closer to one, the segmentation is more accurate. The closer the value is to one, the more accurate the segmentation. The formula for calculation is as follows:

6 $$\begin{aligned} {\text {PA}}=\frac{\sum \nolimits _{i=0}^{k}{{{p}_{ii}}}}{\sum \nolimits _{i=0}^{k} {\sum \nolimits _{j=0}^{k}{{{p}_{ij}}}}}. \end{aligned}$$

Mean pixel accuracy (MPA) is a step up from PA. It calculates PA for each class separately, then averages PA for all classes.

7 $$\begin{aligned} {\text {MPA}}=\frac{1}{k+1}\sum \limits _{i=0}^{k}{\frac{{{p}_{ii}}}{\sum \nolimits _{j=0}^{k}{{{p}_{ij}}}}} \end{aligned}$$

The Jaccard index, or Jaccard similarity coefficient, serves as a statistical measure to assess the similarity and diversity between sample sets. Introduced by Grove Karl Gilbert in 1884, it is formulated as the ratio of verification [78]. The Jaccard coefficient quantifies the similarity of finite sample sets by calculating the size of their intersection divided by the size of their union. This metric is also referred to as Intersection over Union (IoU).

8 $$\begin{aligned}{} & {} J(A,B)=\frac{|A\cap B|}{|A\cup B|}=\frac{|A\cap B|}{|A|+|B|-|A\cap B|} \end{aligned}$$

9 $$\begin{aligned}{} & {} {\text {IoU}}=\sum \limits _{i=0}^{1}{\frac{{{p}_{ii}}}{{{\sum \nolimits _{j=0}^{1}{p}}_{ij}}+ \sum \nolimits _{j=0}^{1}{{{p}_{ji}}}-{{p}_{ii}}}} \end{aligned}$$

The mean intersection over union (mIoU) is used to calculate different categories of IoU in the image, and then calculate the average value is calculated as the final result. For image segmentation, the calculation formula of mIoU is as follows:

10 $$\begin{aligned} {\text {mIoU}}=\frac{1}{k+1}\sum \limits _{i=0}^{k}{\frac{{{p}_{ii}}}{{{\sum \nolimits _{j=0}^{k}{p}}_{ij}}+\sum \nolimits _{j=0}^{k}{{{p}_{ji}}}-{{p}_{ii}}}}. \end{aligned}$$

The Dice coefficient is a fixed similarity measurement function that is commonly used to determine the similarity of two samples. In the segmentation task, we consider the model prediction result and the real mask to be two sets with the same number of elements, and the value of the Dice coefficient is used to judge the quality of the model prediction result.

11 $$\begin{aligned} {\text {DSC}}=\frac{1}{k+1}\sum \limits _{i=0}^{k}{\frac{2\times {{p}_{ii}}}{{{\sum \nolimits _{j=0}^{k}{p}}_{ij}}+\sum \nolimits _{j=0}^{k}{{{p}_{ji}}}}} \end{aligned}$$

The directed average Hausdorff distance from point set *X* to *Y* is given by the sum of all minimum distances from all points from point set *X* to *Y* divided by the number of points in *X*. The average Hausdorff distance can be calculated as the mean of the directed average Hausdorff distance from *X* to *Y* and directed average Hausdorff distance from *Y* to *X*. In the medical image segmentation domain, the point sets *X* and *Y* refer to the voxels of the ground truth and the segmentation, respectively. The average Hausdorff distance between the voxel sets of ground truth and segmentation can be calculated in millimeters or voxels.

12 $$\begin{aligned} AVD=\frac{\frac{1}{X}\sum \limits _{x\in X}{\underset{y\in Y}{\mathop {\min }}\,d(x,y)}+\frac{1}{Y}\sum \limits _{y\in Y}{\underset{x\in X}{\mathop {\min }}\,d(x,y)}}{2} \end{aligned}$$

## Dataset

**Table 1:** Medical image dataset

Datasets	Year	Tasks	Resolving power(pixel)	Sample
STARE [79]	2000	Retinal vascular segmentation	700 $\times$ 605	20
DRIVE [80]	2004	Retinal vascular segmentation	768 $\times$ 584	40
Alizarine [81]	2010	Corneal endothelial cell segmentation	768 $\times$ 576	30
CHASE-DBI [82]	2012	Retinal vascular segmentation	999 $\times$ 960	28
HRF [83]	2013	Retinal vascular segmentation	3304 $\times$ 2336	45
GLAS [72]	2016	Glandular segmentation	567 $\times$ 430 $\sim$775 $\times$ 522	165
MoNuSeg [73]	2017	Nuclear segmentation of multiple organs	1000 $\times$ 1000	30
DSB18 [84]	2018	Nuclear segmentation	$\sim$	670
TNBC [85]	2018	Nuclear segmentation	512 $\times$ 512	50
IDRiD [86]	2018	Segmentation of fundus lesions	4288 $\times$ 2848	516
DDR [87]	2019	Segmentation of fundus lesions	512 $\times$ 512	757
PanNuke [88]	2019	Multiple organ pan cancer cell segmentation	256 $\times$ 256	7904
Brain US [89]	2019	Ventricular septum segmentation	512$\times$ 512	1629
Kvasir-SEG [90]	2020	Gastrointestinal polyp segmentation	332 $\times$ 487$\sim$1920 $\times$ 1072	1000
TM-EM3000 [91]	2021	Corneal endothelial cell segmentation	266 $\times$ 480	184
PROMISE12 [92]	2012	Prostate segmentation	$\sim$	100
BTCV [37]	2015	Abdominal organ segmentation	512 $\times$ 512$\times *$	50
BCV [41]	2015	Abdominal organ segmentation	$\sim$	30
ACDC [93]	2018	Cardiac segmentation	$\sim$	150
BraTS [94]	2018	Brain tumor segmentation	240 $\times$ 240 $\times$ 155	285
MSD [38]	2018	Decathlon Division	$\sim$	2633
LiTS [60]	2019	Liver tumor segmentation	512 $\times$ 512$\times *$	131
KiTS19 [95]	2019	Renal tumor segmentation	$\sim$	210
SegTHOR [43]	2019	Chest organ segmentation	$\sim$	40
Thorax-85 [42]	2021	Chest organ segmentation	$\sim$	85

Unlike general image datasets, medical image annotation requires doctors with professional experience to devote significant time to annotation. The majority of the early pathological image data are of a small scale. Deep learning models, particularly transformer-based models, rely heavily on large-scale data to perform well. A novel labeling strategy involves training a deep learning model with a small amount of data and then manually modifying the model’s prediction results to continuously expand and improve the dataset. Some public datasets used in many popular medical image segmentation tasks have been compiled in Table 1 to assist readers in conducting relevant experiments quickly. In the “Resolving power (pixel)” column of Table 1, “~” indicates that the image resolution in the dataset is not uniform. For example, in the GLAS dataset, the minimum image resolution is 567 × 430 and the maximum resolution is 755 × 522. “*” is only used in 3D image datasets to indicate that the number of channels in the dataset is not fixed, even if the image resolution is the same.

## Summary and outlook

Transformers have emerged as a hot topic in the field of deep learning, and they can be found in a variety of downstream tasks in NLP and computer vision. The hybrid model of the convolutional neural network and transformer performs well in the task of medical image segmentation. However, using transformer to process medical images still presents significant challenges:

1. The medical image dataset is small: labeling medical images requires doctors with professional experience, and medical images have high resolution, so labeling medical images takes time and money. Existing medical image datasets have a small sample size. Using transformers to their full potential in capturing long-distance dependencies necessitates more samples, which most medical image datasets lack.

2. Transformer lacks location information: Object location information is critical for segmentation results in medical image segmentation tasks. Transformer can only embed position information through learning because it does not contain position information. However, the location information is different for different data sets, and the requirements for location information are different, so the methods of learning location are also different, which has a significant impact on the model’s generalization.

3. The self-attention mechanism only works between image patches: after the image is serialized, the calculation of the attention weight is only performed between image patches, and the relationship between the pixels within the image patch is ignored. Critical information between pixels can affect model accuracy when segmenting, recognizing, or detecting small objects and tasks with blurred boundaries.

**Fig. 7:**
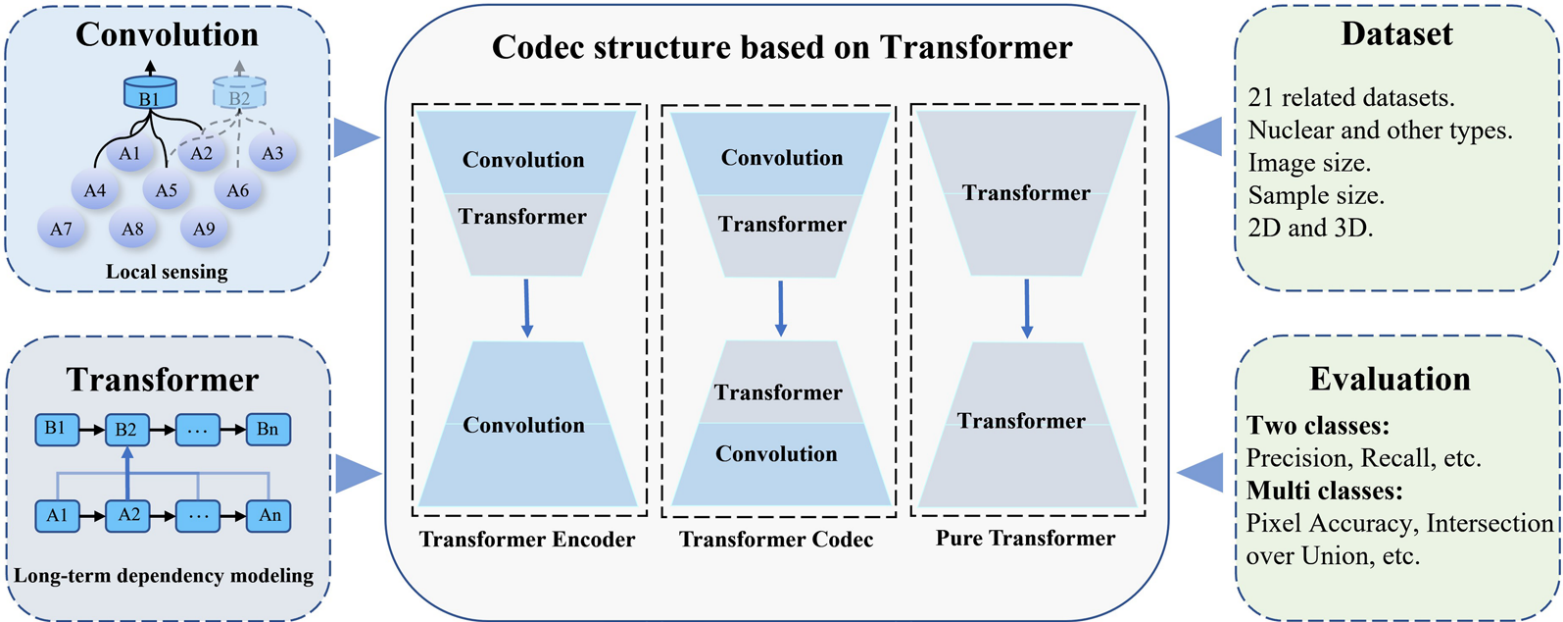
Three network structures for transformer applications in medical image segmentation tasks

Based on the transformer’s current status and challenges in medical image segmentation, the following suggestions and prospects for future research are made:

1. The transformer’s ability to extract global key features from large datasets has been leveraged to train the model on large datasets with auxiliary tasks or to learn existing labeled image features to automatically generate high-confidence pseudo labels. These approaches are effective in addressing the challenge of small-scale medical image datasets.
2. Integrating prior knowledge about the location can assist the model in highlighting important features of the target task. The position encoding for transformer can be thoughtfully designed to incorporate prior knowledge of the image position, thereby enhancing the model’s ability to generalize.
3. Optimizing the model structure is crucial. A large receptive field transformer can extract global key features, while a convolutional neural network is better suited for capturing small local features through continuous convolution pooling, which is essential for segmentation tasks. Therefore, the fusion strategy between the two methods needs to be optimized to fully leverage their respective strengths and ensure the model’s optimal performance.

The transformer has become one of the most popular deep learning frameworks in the last 2 years. It can alleviate the problems of scattered target regions and large shape differences in medical image segmentation tasks due to its advantage of obtaining global context. As shown in Fig. 7, both CNN and transformer have their advantages. The transformer can use the convolutional neural network structure to fully exploit the ability of sample information to extract multiscale local spatial features, allowing the model’s global and local information to achieve a better balance and improve model performance. We summarize recent research on the hybrid model of convolutional neural networks and transformers in this paper. Transformers have good development prospects and high research significance in the field of medical image segmentation, based on the performance of the model in this paper.

## Data Availability

All data can be found on the corresponding page of the cited literature.
